# Microbiota-driven metabolic–immune crosstalk in breast cancer

**DOI:** 10.3389/fphar.2026.1931829

**Published:** 2026-09-01

**Authors:** Jie Guan, Qian Zhou, Jun Chen, Jinnan Zhang, Shuyi Yang, Qingquan Xiong, Jiaan Ye, Yun Zeng, Guang-Hui Ren, Meng Zhang

**Affiliations:** 1 Department of Thyroid and Breast Surgery, Shenzhen Hospital of Southern Medical University, Shenzhen, Guangdong, China; 2 School of Traditional Chinese Medicine, Southern Medical University, Guangzhou, Guangdong, China; 3 Department of Urology Surgery, Shandong Cancer Hospital and Institute, Shandong First Medical University and Shandong Academy of Medical Sciences, Jinan, Shandong, China

**Keywords:** breast cancer, cGAS-STING pathway, glycolysis, immunotherapy, tumor microbiome

## Abstract

Cancer continues to pose a significant risk to women’s health globally, as its progression and treatment resistance arise from an intricate interplay between metabolic reprogramming, immunological evasion, and the tumor microbiome. Augmented glycolysis has emerged as a pivotal element in cancer pathogenesis, altering the tumor microenvironment via the buildup of immune-regulated metabolites that directly influence the activation of the cGAS-STING pathway. This pathway is regarded as a significant modulator of cancer immunity, but new studies indicate that its persistent activation generally results in an immunosuppressive tumor microenvironment instead of robust antitumor immunity. Developments in multi-omics technology have revealed the therapeutic importance of the tumor microbiome, and thus, we focus on recent advancements in tumor microbiota that modify glycolytic pathways to influence the cGAS-STING signaling threshold, thereby impacting cancer immunotherapy.

## 1 Introduction

Breast cancer is the most prevalent malignancy among women globally and the second most common cause of cancer-related mortality (Abedian Kasgri et al., 2022), with significant challenges in its clinical treatment. These issues arise from the intricate, multifactorial interactions within the tumor microenvironment (TME) that existing treatments do not adequately address (Kong et al., 2024). Malignancy is characterized by enhanced glycolysis: in addition to supplying energy for rapid proliferation, glycolytic flux generates acidic metabolites that substantially modify the TME (Ligorio et al., 2025) and facilitate complex interactions between malignant cells and stromal populations, thereby promoting tumor growth, invasion, metastasis, and resistance to treatment (Liu et al., 2024a).

A significant obstacle to effective tumor therapy is immune evasion. The cyclic GMP-AMP synthase (cGAS)–stimulator of interferon genes (STING) pathway, present in both cancerous and immune cells, demonstrates significant functional duality in breast cancer (Wu et al., 2013). Acute activation induces protective antitumor immunity, while prolonged activation paradoxically fosters an immunosuppressive TME and tumor growth (Xiong et al., 2025). The principal glycolytic metabolite, lactate, directly obstructs cGAS-STING signaling, thereby undermining host antitumor immune surveillance (Li et al., 2025a).

At the same time, multi-omics investigations have underscored the clinical importance of the intratumoral microbiota. Unique microbial communities reside in both healthy and malignant breast tissues, with their composition and diversity tightly linked to molecular subtypes and patient outcomes (Fu et al., 2022). Tumor-associated microorganisms and their metabolites alter cancer cell behavior, the immunological environment, and treatment responses via several pathways, including genotoxicity and direct control of glycolytic activity and cGAS-STING signaling. Microbial metabolites, like butyrate and lactate, further refine these pathways (Kaparakis-Liaskos and Ferrero, 2015).

The tumor microenvironment is a complex ecosystem comprising tumor cells, immune cells, and microbial communities. Although previous studies have separately elucidated the roles of glycolysis and the cGAS-STING pathway in tumors, the mechanisms by which microorganisms are involved and the regulatory relationships among these three factors have not yet been systematically elucidated. This article examines the interplay between glycolysis, the cGAS-STING signaling pathway, and the tumor-associated microbiome. Oncogenic microorganisms, acting as upstream regulatory nodes, affect the cGAS-STING signaling threshold by altering the glycolytic flux and thereby affecting the immunological therapeutic response in cancer. The dysregulation of the “microbiota–metabolism–immunity” axis influences the clinical symptoms and therapeutic responses in breast cancer.

## 2 The role of glycolysis in the advancement of breast cancer

### 2.1 Reprogramming the glycolytic function of breast cancer

Glycolysis is a metabolic pathway that converts glucose into lactate to produce energy under hypoxic conditions. The Warburg effect is a hallmark metabolic feature of breast cancer, characterized by the metabolic reprogramming of tumor cells through preferential aerobic glycolysis in an oxygen-rich environment (Icard et al., 2018). This metabolic transformation not only fulfills the energy demands of tumor cell proliferation by rapidly generating ATP, but also provides essential intermediate precursors for the biosynthesis of macromolecules, including nucleotides and amino acids (TeSlaa et al., 2021).

This highly efficient metabolic phenotype does not arise spontaneously but relies on a series of rate-limiting enzymes that are overexpressed and hyperactive. Glucose transporter 1 (GLUT1)-mediated glucose uptake across the membrane is the key rate-limiting step in the sequential catalysis of hexokinase 2 (HK2), phosphofructokinase (PFK), and pyruvate kinase M2 (PKM2), the combination of which promotes a significant increase in glycolytic flux (Ishida et al., 1992; Zaidi and Jaffee, 2019).

In-depth mechanistic studies have revealed that glycolytic enzymes possess non-canonical functions that extend beyond their classical catalytic roles. Specifically, HK2 induces the expression of the immune checkpoint molecule programmed death ligand 1 (PD-L1) by activating the NF-κB signaling axis (Wu et al., 2022), thereby attenuating the cytotoxic effect of CD8^+^ T cells and fostering an immunosuppressive microenvironment (Brown et al., 2002). In contrast, PKM2 acts as a transcriptional coactivator upon nuclear translocation, reshaping the gene expression profile to drive tumor malignancy (Nihira et al., 2025). Fluctuations in its expression levels directly determine the metabolic fate: PKM2 deletion can activate the AMP-activated protein kinase/Kruppel-like factor 4/very long-chain acyl-CoA dehydrogenase (AMPK/KLF4/ACADVL) axis, which promotes a shift from glycolytic to fatty acid oxidation (FAO) metabolism. This finding shows that PKM2 acts as a “molecular switch” that balances glycolysis and lipid metabolism, providing a critical theoretical foundation for explaining the survival strategies of tumor cells in extreme microenvironments (Zaidi and Jaffee, 2019; Luo and Semenza, 2012). Via these “dual” functions, glycolytic reprogramming is integrated with tumor immune evasion mechanisms, underscoring the multifaceted biological effects of metabolic enzymes in tumor progression.

At the systemic regulatory level, multiple upstream signals, including the phosphatidylinositol 3-kinase/protein kinase B/mammalian target of rapamycin (PI3K/AKT/mTOR), hypoxia-inducible factor 1α (HIF-1α), and myelocytomatosis oncogene (MYC), regulate the expression and activity of glycolytic enzymes (Cortellino et al., 2022). Specifically, the PI3K/AKT/mTOR pathway promotes the transcriptional reprogramming of genes involved in glycolysis. A hypoxic microenvironment stabilizes HIF-1α and upregulates GLUT1 and other glycolytic enzymes, while MYC significantly bolsters lactate production by activating lactate dehydrogenase A (LDHA). These regulatory factors act synergistically to establish the metabolic phenotype of breast cancer cells with high rates of glycolysis (Di Biase et al., 2016).

This multifactorial reprogramming of glycolysis exhibits significant metabolic heterogeneity across different molecular subtypes of breast cancer (Xiong et al., 2025), with studies finding that triple-negative breast cancer (TNBC) and human epidermal growth factor receptor 2 (HER2)-positive breast cancer exhibit relatively high glycolytic flux. TNBC cells are particularly dependent on glycolysis, whereas luminal breast cancer exhibits a relatively stable metabolism. Such differences in subtype-specific metabolic profiles provide a critical theoretical foundation for precise metabolic intervention.

#### 2.1.1 Regulatory mechanisms governing tumor growth and metastasis

As the central metabolic engine satisfying both energetic and biosynthetic demands, glycolysis mediates multiple pathways that propel cancer malignancy. These tumor-promoting effects are governed by complex regulatory networks and exert their effects at multiple levels, including the transcriptional, post-transcriptional, and post-translational levels.

At the transcriptional level, HIF-1α is a key transcription factor driving the expression of glycolytic genes, and its activity is regulated by the PI3K/AKT/mTOR pathway. In TNBC, the activation of HIF-1α directly upregulates the expression of GLUT1, HK2, and LDHA, thereby establishing a sustained high glycolytic flux under hypoxic stress to support rapid tumor cell proliferation. Additionally, the oncogenic transcription factor MYC activates LDHA through a pathway independent of HIF-1α, further enhancing lactate production; this feature is particularly prominent in MYC-amplified breast cancer.

At the post-transcriptional level, long non-coding RNA (lncRNA) serves as a key factor in maintaining a high-throughput intracellular glycolysis state. For example, the lncRNA HANR can regulate post-translational rate-limiting enzymes, directly bind to and antagonize the HK2 proteasome degradation pathway, and maintain sustained high-throughput intracellular glycolysis in TNBC (Ishida et al., 1992). Similarly, the lncRNA NEAT1, which is upregulated in aggressive subtypes of breast cancer, acts as a scaffold to facilitate the assembly of the PGK1/PGAM1/ENO1 glycolytic enzyme complex, thereby enhancing substrate channel efficiency and increasing the proliferative and metastatic potential of tumor cells. In addition, in metastatic non-small cell lung cancer, specific lncRNAs act as competitive endogenous RNAs by binding to miR-1285-3p to mitigate its effects on glucose-6-phosphate isomerase (G6PI), an enzyme implicated in TNBC development whose post-transcriptional repression significantly increases glucose uptake and lactate production (Liu et al., 2024a). By reshaping the cellular metabolic environment through the aforementioned mechanisms, this significantly enhances the proliferative capacity and metabolic adaptability of cancer cells, thereby promoting tumor initiation, growth, and metastasis (Park et al., 2021).

At the post-translational level, the non-classical functions of glycolytic enzymes amplify their carcinogenic effects. Following nuclear translocation, PKM2 acts as a transcriptional coactivator, reshaping the gene expression profile to drive tumor progression (Nihira et al., 2025). Post-translational modifications of PKM2 play a key role in its functional regulation: Ser37 phosphorylation induced by growth factors promotes the nuclear translocation of PKM2, while Tyr105 phosphorylation inhibits its enzymatic activity and supports anabolic reprogramming. In addition, modifications such as acetylation and O-GlcNAc glycosylation further influence the oncogenic function of PKM2 by regulating its oligomerization state and subcellular localization (Prakasam et al., 2018).

In addition, the metabolic byproducts produced by glycolysis also have a direct tumor-promoting effect. Methylglyoxal can covalently modify and inactivate breast cancer susceptibility protein 2 (BRCA2), thereby disrupting DNA damage repair mechanisms, exacerbating genomic instability, and accelerating the accumulation of oncogenic mutations (Kong et al., 2024). This mechanism is particularly associated with hereditary breast cancer.

Beyond the intracellular regulatory cascades described above, elevated glycolytic activity is significantly positively correlated with poor clinical prognosis and the risk of metastasis. Ligorio et al. reported that early downregulation of intratumoral glycolytic activity is closely associated with favorable therapeutic responses in patients, providing further evidence that a high-glycolytic-flux phenotype promotes tumor growth and distant metastasis. Mechanistically, this metabolic preference not only confers a growth advantage on cancer cells in complex microenvironments but also provides the necessary metabolic foundation for the epithelial–mesenchymal transition (EMT) and subsequent hematogenous dissemination by enhancing lineage plasticity (Cossu et al., 2023).

#### 2.1.2 Reshaping cancer cell properties and TME

The biological effects of glycolysis in breast cancer extend beyond intracellular energy metabolism; through the extracellular transport of metabolites, it reshapes the physicochemical properties and cellular composition of the tumor microenvironment (TME). Due to the excessive accumulation of lactate, driven by high glycolytic flux, the TME becomes significantly acidified, a well-documented phenomenon in other solid tumors. For example, in liver cancer, Liu et al. found that GJB2 upregulates PD-L1 expression by activating the glycolysis-NF-κB pathway and inducing immune evasion (Liu et al., 2024b). However, in lung adenocarcinoma, differential expression of the rate-limiting glycolytic enzyme fructose-1,6-bisphosphatase (FBP1) influences extracellular acidification and DNA damage responses by regulating lactate levels (Li et al., 2025b). This cross-cancer evidence reveals that the NF-κB/PD-L1 axis is activated by glycolysis, demonstrating crosstalk between metabolic reprogramming and immune evasion. In breast cancer, the coupling effects of metabolism and immunity exhibit significant heterogeneity, especially in the highly glycolytic subtype.

In-depth mechanistic studies have shown that the non-metabolic functions of rate-limiting enzymes in glycolysis play a critical role in remodeling the malignant phenotype of cancer cells. At the epigenetic level, RNA modifications play a central role. For example, m6A methyltransferase 14 (N6-methyladenosine, m6A) stabilizes the long non-coding RNA LINC01094 by acting as a “molecular scaffold” to recruit the PKM2/Jumonji domain-containing protein 5 (JMJD5) complex, inducing PKM2 nuclear translocation, activating the HIF1-α/β-catenin signaling pathway, and thereby driving glycolytic reprogramming and malignant proliferation in breast cancer cells (Li et al., 2024). PKM2 can form a transcriptional inhibitory complex with the epigenetic factor enhancer of zeste homolog 2 (EZH2), while silencing the carnitine transporter solute carrier family 16 member 9 (SLC16A9) blocks the FAO pathway at the transcriptional level. Notably, TNBC exhibits extreme metabolic plasticity. Even when the glycolytic pathway is blocked, cancer cells can flexibly switch to the FAO functional mode by activating latent plasticity, thereby alleviating microenvironmental stress (Zhang et al., 2024). This metabolic adaptation mechanism confers a survival advantage to cancer cells in extreme environments, ultimately driving the malignant progression of tumors and treatment resistance. In addition to FAO, breast cancer cells can also respond to the metabolic stress caused by glycolysis inhibition through other alternative pathways. Among these, glutaminolysis is an important compensatory metabolic pathway in TNBC. When glycolysis is impaired, tumor cells can convert glutamine into glutamate by upregulating the expression of glutamine transporters and glutaminase (GLS), and further generate α-ketoglutarate (α-KG) to replenish the tricarboxylic acid cycle, thereby maintaining mitochondrial respiration and ATP supply. In addition, the activation of the *de novo* synthesis pathway for serine and glycine provides an alternative source of nucleotides and NADPH (Zou et al., 2025). However, in order to survive under metabolic stress when glutamine metabolism is impaired, cancer cells adapt to the harsh environment by activating the serine synthesis pathway (SSP) via the AMPK molecule. Through PSAT1, a key enzyme in this pathway, they continue to produce α-KG to support the TCA cycle, thereby ensuring the survival and proliferation of cancer cells (Qiu et al., 2024). To systematically integrate these pharmacological agents, Table 1 provides a comprehensive cross-comparison of their molecular and cellular activities, *in vivo* antitumor efficacy, clinical development stages, and multimodal combination strategies.

**TABLE 1:** Pharmacological characteristics, translational landscape, and combination strategies for targeted therapy in breast cancer.

Target and pathway	Therapeutic agents and interventions	Cellular activity	*In* *vivo* anti-tumor efficacy	Strategy and translational stage	Combination potential and synergistic strategies
Glycolysis monotherapy targets					
GLUT1	Radixins, NV-5440, SFT-31, WZB117	Block glucose transmembrane transport; deprive tumor cells of their carbon supply at the source	Impair proliferation and survival potential of tumor cells	Preclinical	Synergizes with anti-PD-L1 to enhance immune cell infiltration; combined with cisplatin
HK2	2-DG, 3-BrPA	Inhibit rate-limiting enzyme activity; non-canonically induce PD-L1 via the NF-kB axis	Reduce intratumoral glycolytic activity and ATP production	Phase I/II clinical trial	Combine with metformin to synergistically enhance sensitivity of drug-resistant cells to GLUT1 inhibitors
PFKFB3	YN1, 3-PO, KN0438757, N4A	Suppress PFKFB3 enzyme synthesis and disrupt cellular flux to induce synthetic lethal effects	Suppress glucose metabolism and growth of breast cancer cells	Preclinical/Early phase I	Synergize with immune checkpoint blockades and chemotherapy
PKM2	Alkanin, TRIM35, shikonin	Regulate tetramer/dimer balance; act as a molecular switch between glycolysis and FAO.	Inhibit tumor growth, malignant behavior, and distant metastasis	Preclinical	Combine with paclitaxel or immune checkpoint blockades; PKM2 deletion activates AMPK/KLF4/ACADVL axis
LDHA	FX11, galloflavin, NHI	Inhibit lactate synthesis, reduce accumulation of metabolic byproducts, and enable immune cell infiltration	Reduce tumor progression and restore cellular homeostasis	Preclinical	Enable immune cell infiltration, restore TME pH, derepress STING signaling, and sensitize anti-PD-L1 therapy
cGAS-STING pathway interventions					
STING agonists	Small-molecule STING agonists, CDNs, manganese-based nano-agonists	Directly activate STING, induce Type-I IFNs, and reverse the “cold” tumor phenotype	Reverse cold tumor phenotype and improve immunotherapy responsiveness	Translational/Phase I/II	Synergize with immune checkpoint blockades (anti-PD-l1) and radioimmunotherapy
Systemic metabolism & microbial interventions					
Systemic metabolism	Fasting-mimicking diet (FMD)	Reconfigure tumor metabolic dependencies globally and downregulate intratumoral glycolytic activity	Globally downregulate intratumoral glycolytic activity	Phase I/II clinical trial	Combine with neoadjuvant chemotherapy to significantly improve pathological complete response (pCR) rate
Microbial dysbiosis	Antibiotics, probiotics (*Lactobacillus* spp.)	Clear pathogenic intracellular colonizers or redirect glucose metabolism toward glycolysis	Slow pulmonary metastasis with minimal impact on primary tumor	Preclinical & clinical translation	Combine with metabolic inhibitors or chemotherapy to prevent postoperative metastasis and recurrence
Multimodal combination strategies					
cGAS-STING + immune checkpoint	Mn^2+^based silica nanocarriers + anti-PD-l1 antibody	Target mitochondria to induce mtDNA release in synergy with Mn^2+^	Drive STING activation and enhance anti-PD-L1 therapeutic efficacy	Combination immunotherapy	Transform TME from “cold” to “hot” and sensitize anti-PD-l1 non-responsive tumors
STING activation + lactate consumption	Bio-mineralized engineered OMVs-ECL	OMVs and Mn^2+^ induce STING phosphorylation while loaded LDH consumes lactate to lift acid stress	Lift immunosuppressive barrier and restore local antitumor immunity	Multi-functional nano-agonists	Dual-action strategy: Synergize lactate metabolism modulation with cGAS-STING activation
BRCA2/HR pathway (DNA damage repair)	GDP-mannose	Blocks BRCA2-mediated HR repair, causing mtDNA accumulation to activate downstream STING signals	Induces DNA damage and mtDNA accumulation to trigger STING	Metabolic-sensitized immunotherapy	Dominant regulatory role in immunotherapy of TNBC and BRCA2-proficient cancers
MCT4 transporter & metabolic crosstalk	Sodium butyrate + 3-bromopyruvate (3-BP)	Microbial metabolite butyrate induces MCT4 expression in tumor cells	Significantly enhances the antitumor activity of 3-BP	Microbiome-metabolism combination	Utilize microbial metabolites to sensitize tumor cells to rate-limiting enzyme inhibitors
Metabolic plasticity (FAO and epigenetics)	EZH2 inhibitor + FAO inhibitor	Block adaptive metabolic shift from glycolysis to FAO, preventing survival	Induce metabolic crisis and eliminate TNBC cells	Dual metabolic blockade	Eliminate TNBC cell populations that have undergone metabolic conversion

2-DG, 2-deoxy-D-glucose; 3-BP, 3-bromopyruvate; 3-BrPA, 3-bromopyruvate; CDNs, cyclic dinucleotides; EZH2, enhancer of zeste homolog 2; FAO, fatty acid oxidation; FMD, fasting-mimicking diet; GLUT1, glucose transporter 1; HK2, hexokinase 2; HR, homologous recombination; LDH, lactate dehydrogenase; LDHA, lactate dehydrogenase A; MCT4, monocarboxylate transporter 4; NF-κB, nuclear factor kappa B;OMVs, engineered bacterial outer membrane vesicles; PD-L1, programmed death ligand 1; PFKFB3, 6-phosphofructo-2-kinase/fructose-2,6-biphosphatase 3; PKM2, pyruvate kinase M2; STING, stimulator of interferon genes; TME, tumor microenvironment; TNBC, triple-negative breast cancer.

### 2.2 Glycolysis-mediated drug resistance to cancer therapies

An abnormally elevated glycolytic flux is one of the key drivers of drug resistance in breast cancer. This metabolic shift not only provides cancer cells with an emergency energy reserve under drug pressure but also builds a natural resistance barrier by reshaping intracellular REDOX homeostasis. Specifically, high glycolytic activity optimizes the antioxidant defense system through directed metabolic flux, effectively scavenging chemotherapy-induced reactive oxygen species (ROS) (Dai et al., 2020; Qing et al., 2020) and thereby significantly attenuating drug cytotoxicity. At the same time, the accumulation of lactate and extracellular acidification resulting from upregulated glycolysis not only inhibits the transmembrane uptake of chemotherapeutic agents through a physical barrier (Liu et al., 2024a) but also drives the enrichment of Treg cells and upregulation of PD-L1 expression, thereby establishing a robust immune evasion barrier within the TME (Zhang et al., 2023).

In addition, the non-metabolic functions of rate-limiting enzymes play a critical role in drug resistance, which can be reversed by targeting glycolysis, as demonstrated in research by Niu et al. (2025); this mechanism involves the abnormal activation of key enzymes and their regulatory factors, driving cells into an anti-apoptotic steady state, along with the synergistic activation of signaling pathways such as PI3K/AKT/mTOR (Niu et al., 2025). Metabolic plasticity is also an important mechanism underlying drug resistance. In platinum-resistant cancer cells, metabolic patterns often undergo adaptive shifts, transitioning from glycolysis to fatty acid uptake and β-oxidation to maintain survival under cisplatin-induced oxidative stress (Tan et al., 2022).

The targeting of glycolysis has evolved from early “substrate competition” strategies to precise interventions in rate-limiting enzyme activity and metabolic signaling networks, emerging as a key Frontier in overcoming drug resistance.

In the initial stages of glycolysis, high GLUT1-mediated glucose uptake is critical for maintaining the metabolic advantage of tumor cells. A series of GLUT1 inhibitors (such as radixins, NV-5440, SFT-31, and WZB117) cut off the supply of carbon sources to tumor cells by blocking the transmembrane transport of glucose, thereby depriving them of their ability to proliferate and survive at the source (M et al., 2014; Xintaropoulou et al., 2015). At the level of rate-limiting enzyme regulation, small-molecule HK inhibitors (such as 2-DG and 3-BrPA), when used in combination with metformin, significantly and synergistically enhance the sensitivity of resistant cells by disrupting early glucose phosphorylation (Ilegems et al., 2022). PFKFB-3 inhibitors (such as 3-PO, KN0438757, and N4A) and PKM2 inhibitors that target the final step (such as Alkanin, TRIM35, and shikonin) induce synthetic lethality in tumor cells by disrupting cellular metabolic flux (O'Neal et al., 2016). In addition, LDHA, a key enzyme that catalyzes the conversion of pyruvate to lactate, has emerged as a new target for cancer therapy. LDHA inhibitors (such as FX11, gallaxanthin, and NHI) can reduce the accumulation of metabolic byproducts, promote immune cells infiltration, and restore cellular homeostasis, thereby exerting antitumor effects (Farabegoli et al., 2012; Le et al., 2010).

At the systemic regulatory level, HIF-1α, as a central transcription factor in metabolic reprogramming, not only directly promotes the expression of genes related to glycolysis but also inhibits oxidative phosphorylation and the tricarboxylic acid cycle, thereby preserving the “Warburg phenotype” in tumor cells and promoting drug resistance (Icard et al., 2018). Therefore, small-molecule inhibitors or degradation strategies targeting HIF-1α are important potential approaches to overcome drug resistance in breast cancer.

It is worth noting that macroscopic metabolic interventions can act synergistically with microscopic metabolic targeting. A clinical trial involving TNBC patients demonstrated that a fasting simulated diet (FMD) combined with neoadjuvant chemotherapy significantly improved the pathological complete response rate (Salvadori et al., 2021). This suggests that altering the systemic nutritional environment can globally reconfigure tumor metabolic dependencies, thereby enhancing therapeutic efficacy and reducing the risk of drug resistance. In summary, a multi-tiered intervention strategy targeting glycolysis—ranging from the inhibition of GLUT1-mediated glucose uptake and suppression of rate-limiting enzymes such as HK2/PFKFB3/PKM2/LDHA to HIF-1α-driven transcriptional reprogramming, as well as systematic metabolic interventions based on fasting-mimicking diets—provides a comprehensive pathway from molecular targets to clinical translation for reversing breast cancer resistance, and is expected to advance the realization of multidimensional combined therapeutic strategies integrating “metabolism, immunity, and the microenvironment”.

It is worth noting that glycolytic reprogramming is not a spontaneous process in tumor cells, but is profoundly influenced by the microbial communities and their metabolites in the tumor microenvironment. Recent studies have shown that short-chain fatty acids of microbial origin (such as butyrate) can inhibit glycolytic flux by suppressing histone deacetylase activity, thereby altering the acetylation status of rate-limiting enzymes in glycolysis (HK2 and PKM2) (Rodrigues et al., 2015). In addition, lactate can maintain the expression and enzymatic activity of the rate-limiting enzymes of glycolysis—hexokinase (HK) and phosphofructokinase (PFK)—by activating its receptor, HCAR1, thereby sustaining the glycolytic flux in breast cancer cells. Knocking out HCAR1 reduces the expression and activity of HK and PFK and shifts cellular metabolism from glycolysis to oxidative phosphorylation (Jin et al., 2022). These findings suggest a close functional link between the tumor microbiome and glycolytic metabolism. The following sections will further explore how the tumor microbiome influences the cGAS-STING pathway by regulating glycolytic metabolism.

## 3 Effects of the cGAS/STING signaling pathway on breast cancer

The malignant progression of breast cancer is not only dependent on metabolic reprogramming but is also restricted by innate immune surveillance (Xu et al., 2025). Among these mechanisms, the cGAS-STING pathway, as a key cytoplasmic DNA-sensing system, plays a dual role in tumor immunity and TME remodeling, with high background dependence (Ni et al., 2022). Acute activation can stimulate effective antitumor immune responses and provide new opportunities for immunotherapy, but prolonged and persistent stimulation induces an immunosuppressive microenvironment and drug resistance.

Taking TNBC as an example, it is the most immunogenic subtype of breast cancer. A high density of tumor-infiltrating lymphocytes (TILs) is commonly observed in the TME of TNBC, indicating that the immune system has been activated and recruited to the tumor site. However, the TME in TNBC also exhibits complex immunosuppressive characteristics: M2-type tumor-associated macrophages (TAMs) infiltrate the tumor and exert immunosuppressive effects; Regulatory T cells (Tregs) and myeloid-derived suppressor cells (MDSCs) accumulate in large numbers and, by secreting inhibitory cytokines such as IL-10 and TGF-β to suppress antitumor immunity; Tumor cells themselves directly suppress T cell activity by upregulating immune checkpoint molecules like PD-L1 (Guo et al., 2024). This complex feature of having high immunogenicity alongside immune suppression profoundly affects the occurrence, development, and treatment response of TNBC. Therefore, understanding the regulatory mechanisms of innate immune recognition and adaptive immune activation in TNBC is crucial for treatment.

Within this regulatory network, the cGAS-STING pathway serves as a bridge connecting the innate and adaptive immune systems. In dendritic cells (DCs), STING activation promotes their maturation and antigen cross-presentation, thereby priming tumor-specific T cells. However, for immunosuppressive cell populations, STING-mediated regulation exhibits dynamic phase-dependent characteristics: acute activation promotes the repolarization of antitumor M1-type TAMs and attenuates Treg activity, whereas chronic activation promotes M2-type TAM polarization, Treg accumulation, and MDSC expansion (Ni et al., 2022; Zhang and Huang, 2025; Sun et al., 2025). These functional characteristics, which depend on cell type and duration of action, directly determine whether STING acts to eliminate tumors or induce immune tolerance (Lu et al., 2026).

### 3.1 Mechanisms that cause tumor cell death

The cGAS-STING pathway serves as a central bridge linking innate and adaptive immunity. Under the classical activation pathway, activation of the cGAS-STING pathway induces a burst of Type I interferon (IFN-I) secretion, thereby activating cytotoxic CD8^+^ T cells, enhancing antigen presentation, and promoting an adaptive immune response (Ablasser and Gulen, 2016; Zhang et al., 2020; Liang et al., 2021). It particularly effectively enhances the immunogenicity of TNBC, reverses the cold tumor phenotype, and improves the effect of immunosuppressants (Xu et al., 2024).

Given the complex landscape of immunotherapy for breast cancer, the development of new biomarkers and targeted delivery systems capable of responding to these biomarkers has become a key direction in the field (Hoong et al., 2020). In addition, remodeling of the microenvironment from an “immunotatic” to a “T cell inflammatory” state has become a core feature of STING agonists. Sun et al. (2025) further emphasize that the development of novel delivery systems and combination therapies will help achieve controlled activation of the STING pathway in breast cancer, thereby reducing the risk of systemic toxicity and overcoming the immunosuppressive TME (Sun et al., 2025). The latter, alongside enhancing drug permeability, is a core challenge in TNBC treatment. In recent years, nanomaterials have emerged as a research hotspot due to their ability to be precisely delivered to the target site and their multifunctional properties (Li et al., 2025c). Nanocarriers that modulate the cGAS-STING signaling pathway can reshape the TME, overcoming traditional drugs’ limitations of poor permeability and low response rates (Wang et al., 2024; Pindiprolu et al., 2025). The self-oxygenating nanoradiation radiosensitizer ALFM, developed by Wang et al., enhances antitumor immune responses by activating ICD and the cGAS-STING signaling pathway, thereby improving radioimmunotherapy for TNBC (Zhang and Huang, 2025).

### 3.2 Drive invasion and distant metastasis

In addition to the acute immunostimulatory effects described above, the biological function of the cGAS-STING pathway is highly dependent on the temporal and spatial context in which it occurs. Short-term immune activation can stimulate antitumor immunity, while persistent chronic inflammatory stimulation can lead to immune exhaustion and promote tumor progression, which is one of the reasons why most STING agonists fail in clinical trials (Cossu et al., 2023).

Persistent endogenous DNA leakage caused by genomic instability induces the activation of the non-canonical NF-κB signaling pathway, leading to the sustained release of inflammatory cytokines such as IL-6 and TNF. This prolonged inflammatory environment maintains a state of chronic inflammation and induces the epithelial–mesenchymal transition (EMT) in cancer cells, enhancing their invasive capacity. Tumors often exploit metabolic programming to evade the body’s immune surveillance. In TNBC, accumulated fumarate directly inactivates the STING protein through succinylation. Simultaneously, high glucose flux activates the NSUN2-TREX2 axis, accelerating cytoplasmic DNA degradation and leading to the depletion of sensor substrates, thereby allowing tumors to evade recognition by the innate immune system. Intracellular bacteria trigger the accumulation of cytoplasmic dsDNA, further activating the STING-mediated IL-17B pathway, which induces neutrophils to suppress killer T cells. This immunosuppressive environment directly leads to the spread, recurrence, and distant metastasis of cancer cells.

However, clinical translation remains a challenge (Li et al., 2022). Lu et al. systematically analyzed the key obstacles to targeting this pathway. Due to drug delivery, tumor heterogeneity, and the dual polarization effect. First, the endogenous agonists of STING, cyclic dinucleotides (CDNs), are highly polar and have extremely short half-lives—clinical data show that the terminal half-life of ADU-S100 in humans is only about 24 min, and that of MK-1454 is only about 1.5 h. Because they struggle to cross transport barriers, it is difficult to maintain effective concentrations within tumors. Second, significant inter-tumor and intra-tumor heterogeneity leads to vast differences in how different lesions respond to STING agonists (Lu et al., 2026). To address CDN delivery bottlenecks, researchers have developed a lipid nanoparticle system (LNP-B) modified with pH-sensitive polycationic polymers for intravenous administration of CDN. This system significantly prolongs the circulating half-life of CDN, enhances its accumulation in tumors, the spleen, and tumor-draining lymph nodes, and effectively triggers a STING-mediated antitumor immune response (He et al., 2025). Meanwhile, small-molecule agonists face issues related to nonspecific distribution and off-target effects. Research suggests that harnessing the immunological precision of the STING pathway and maintaining optimal immune conditions in terms of timing and stimulus intensity requires the synergistic regulation of intelligent nanodelivery materials, agonists, and chemotherapy (Sun et al., 2025).

### 3.3 Differences in metabolic and immune properties of breast cancer subtypes

Breast cancer is not a single disease. Its different molecular subtypes exhibit significant heterogeneity in terms of cGAS-STING status, molecular regulation, clinical impact, and immunological outcomes (Hines et al., 2023; Ignatiadis and Sotiriou, 2013), with the degree of metabolic remodeling being positively correlated with tumor invasiveness (Jungles et al., 2022; Feng et al., 2018). Importantly, this subtype-specific heterogeneity also extends to glycolysis dependence and intratumoral microbiome characteristics: TNBC exhibits extremely high glycolytic flux accompanied by severe dysbiosis, whereas the Luminal and HER2+ subtypes display distinct glycolytic dependence and unique microbial diversity. To provide a more intuitive comparison of biological characteristics across subtypes, Table 2 systematically summarizes the glycolytic preferences, intratumoral microbiome profiles, cGAS-STING status, and immunological outcomes of ductal breast cancer, HER2-positive breast cancer, and triple-negative breast cancer.

**TABLE 2:** Glycolytic activity, intratumoral microbiota, and cGAS-STING profiles across breast cancer subtypes.

Subtypes	Glycolytic activity	Intratumoral microbiota features	cGAS-STING status and molecular mechanisms	Clinical impact and immunologic outcomes
TNBC	High glycolytic flux and high dependency; G6PI/HANR maintain high-throughput glycolysis, yielding high lactate levels	Enriched with *Fusobacterium* *nucleatum*; intracellular colonization induces ADSL phosphorylation and fumarate accumulation	Under metabolic repression, the accumulated metabolite fumarate inactivates STING through succinylation, thereby facilitating the degradation of cytoplasmic DNA in coordination with the NSUN2 axis	A state of high immunosuppression
Luminal type	Relatively low/Stable; lower glycolytic dependency compared to TNBC/HER2+	Highest alpha-diversity; dominated by commensal taxa such as *Staphylococcus* *epidermidis* and *Lactobacillus* species	This is a persistent chronic activation state, primarily associated with cellular stress and metabolic stress induced by long-term hormone therapy	Immune escape and tolerance: Inducing T cell infiltration and reducing sensitivity to targeted therapy
HER2+ type	Moderate to high; elevated glycolytic flux regulated by rate-limiting enzymes	Intermediate diversity; enriched with ralstonia and proteobacteria interacting with HER2 signaling	This manifests as a chronic state of cellular exhaustion characterized by pathway inactivation, which is associated with HER2-mediated signaling interference	Establishment of an immune barrier: Maintaining low-grade chronic inflammation and inducing T cell aggregation

ADSL, adenylosuccinate lyase; cGAS, cyclic GMP-AMP synthase; G6PI, glucose-6-phosphate isomerase; HANR, HCC-associated long non-coding RNA; HER2+, human epidermal growth factor receptor 2-positive; Luminal, hormone receptor-positive breast cancer; NSUN2, NOP2/Sun RNA methyltransferase 2; STING, stimulator of interferon genes; TNBC, triple-negative breast cancer.

## 4 Breast cancer “microbiome–metabolism–immunity” axis

### 4.1 Microbe-driven genome and spatial remodeling

#### 4.1.1 Genetic damage and skeletal recombination

During tumor progression, tumor cells interact with neighboring cells to form a TME that supports tumor growth. Alongside the host cells, the tumor microbiome is present in breast cancer tissue and promotes tumorigenesis and malignant progression through various mechanisms. These interactions involve the regulation of gene expression in neighboring cells, thereby altering their function.

First, at the genetic level, the tumor microbiome can induce genetic damage, laying the foundation for tumorigenesis. Through multi-level mechanisms ranging from genetic toxicity to spatial migration, microbes can produce various toxins that directly damage host cell DNA or indirectly damage mtDNA by inducing ROS, thereby activating the cGAS-STING pathway. These damages can lead to the inactivation of tumor suppressor genes (such as TP53) or the activation of oncogenes, providing a genetic basis for tumorigenesis (Sepich-Poore et al., 2021).

Second, at the signal transduction level, microorganisms induce abnormal cell proliferation by secreting metabolites or effector molecules (such as CATA and AVRA). For example, they activate the Wnt/β-catenin signaling pathway within host cells, promoting abnormal cell growth, which is closely associated with the initiation, growth, and resistance of breast cancer (Xu and Wang, 2025).

Finally, at the metastasis level, the tumor microbiome also plays a significant promotional role. Staphylococci, Lactobacilli, Streptococci, and other microorganisms within breast cancer tissues can modulate the cytoskeleton, helping tumor cells resist mechanical vascular stimulation and thereby promoting hematogenous metastasis. Clearing bacteria from the tumor significantly slows lung metastasis in breast cancer while having a minimal impact on the primary tumor, indicating that intratumor microorganisms play a specific role in regulating tumor metastasis (Fu et al., 2022).

The intratumoral microbiome in breast cancer, while exerting local effects, constitutes a distinct new field of biology. Sepich-Poore et al. (2021) provided a systematic review of the correlation between the microbiome and human cancer in *Science*, proposing that the tumor microbiome represents a new biological discipline (Chen et al., 2023a). Xu and Wang (2025) focused on the microbiome in breast cancer, providing a detailed overview of knowledge ranging from basic research to clinical translation. While both healthy and cancerous breast tissue contain microorganisms, their compositions differ significantly. Tumor-associated microbiota exert functional mechanisms such as genotoxicity, activation of the Wnt/β-catenin pathway, and regulation of inflammation and immunity, and assist tumor cells in resisting blood flow shear stress. Furthermore, the abundance of different microorganisms varies across breast cancer subtypes; for example, in TNBC, *Fusobacterium nucleatum* is enriched and exhibits distinct subtypes (Xu and Wang, 2025). In contrast, non-TNBC subtypes exhibit a unique intratumoral microbiome profile that corresponds to their biological characteristics. Luminal-type breast cancer is characterized by the highest α-diversity in the microbiome, dominated by commensal strains such as *Staphylococcus epidermidis* and *Lactobacillus* (Xu and Wang, 2025). At the same time, HER2-positive breast cancer exhibits moderate levels of microbial diversity, characterized by an enrichment of lineages such as the genus Ralstonia and the phylum Proteobacteria; these bacteria can activate host Toll-like receptors (TLRs), thereby acting synergistically with the HER2-downstream PI3K/AKT and MAPK oncogenic pathways to promote tumor cell proliferation and metabolic adaptation (Nejman et al., 2020).

#### 4.1.2 Intracellular colonization and immune shielding

In addition to these general interactions, the immunological consequences of microbial presence are critically determined by the spatial context. Bacteria that stably colonize tumor cells not only promote metastasis through mechanical regulation but may also help tumors evade immune clearance by inhibiting the cGAS-STING pathway or remodeling glycolytic metabolism (Yao et al., 2026).

At the level of the immune TME, the tumor microbiome creates a local barrier that promotes tumor progression by inducing chronic inflammation and suppressing antitumor immunity. Host pattern recognition receptors (PRRs) recognize intratumoral microbiota as pathogen-associated molecular patterns (PAMPs). The microbiota continuously activates key inflammatory signaling pathways, such as NF-κB, releases large amounts of proinflammatory cytokines and chemokines, and establishes a chronic inflammatory cycle. Furthermore, bacterial components in the microenvironment may abnormally activate the complement system, exacerbating local inflammation, suppressing antitumor immune responses, and promoting angiogenesis and tissue invasion (Sepich-Poore et al., 2021). Intracellular bacteria trigger the accumulation of cytoplasmic dsDNA, activate the cGAS-STING and Interleukin 17B (IL-17B) pathways, induce neutrophil immunosuppression, suppress killer T cells, and promote cancer cell metastasis and recurrence. Meanwhile, under hypoxic and high metabolic stress induced by *Fusobacterium* nucleatum, intracellular bacterial colonization induces phosphorylation of adenylosuccinate lyase (ADSL), leading to abnormal accumulation of its metabolite, fumarate, which inhibits STING protein function through succinylation modification. At the molecular level, tumor cells fully evade immune surveillance signals (Duan et al., 2025).

### 4.2 Glycolysis-mediated inhibition of cGAS-STING recognition

The involvement of glycolysis in the cGAS-STING pathway exhibits strong context dependence and functional polarization. One mechanism involves the glucose–NSUN2–TREX2 axis, through which epigenetic modifications and nuclease activity are combined to pathologically suppress STING activation. Conversely, in specific immune cells, metabolic flux can provide energy support to dendritic cells, thereby facilitating STING-mediated immune responses.

Although evidence demonstrating that the microbiota directly drives glycolytic reprogramming in breast cancer is still emerging, the two exhibit a high degree of functional coupling, jointly shaping an immunosuppressive metabolic environment. Research by Li et al. (2025a) revealed the relationship between microbial metabolites and glycolysis, and direct evidence from recent studies has demonstrated that intervention with a multi-strain probiotic consortium (predominantly *Lactobacillus* species) can reshape breast tissue metabolism by redirecting glucose metabolism toward glycolysis (Arnone et al., 2025). This microbial-driven metabolic reprogramming alters the expression of key glycolytic enzymes and metabolic intermediates, providing biochemical substrates—such as lactate, fumarate, and other glycolytic byproducts—the ability to fine-tune cGAS-STING activity. In this microbiotic-driven high-glucose environment, intracellular glucose mechanisms are abnormally activated, and glucose flux determines the starting point for immune evasion through epigenetic mechanisms. Furthermore, high intracellular glucose levels suppress cGAS-STING signaling by activating the RNA methyltransferase NSUN2, which stabilizes TREX2 mRNA and accelerates the degradation of tumor-derived DNA within the cell. This is a key driver of tumor progression and influences its response to PD-L1 therapy (Chen et al., 2023b).

As a key metabolite synthesized by microorganisms, butyrate possesses multifaceted regulatory capabilities. Microbially synthesized butyrate esters act as natural histone deacetylase (HDAC) inhibitors and exhibit significant epigenetic remodeling capabilities, and butyrate can inhibit glycolysis in cancer cells by altering the acetylation status of key activator domains in critical glycolytic enzymes (Rodrigues et al., 2015). Furthermore, studies have shown that butyrate possesses profound signaling regulatory capabilities, targeting the Wnt/β-catenin signaling axis, which is a central driver of metabolic reprogramming in breast cancer, and butyrate can also suppress this pathway to reduce glycolytic activity (Liang et al., 2023; Kharazi et al., 2025). This finding provides a reference for how the tumor microbiome regulates glycolysis through metabolites, although the underlying mechanisms still require validation through rigorous experimental studies.

Microbial colonization exacerbates hypoxia and acidification in the TME, establishing a multi-layered systemic immune barrier and precisely regulating the immune-sensing threshold of the body. Hypoxic stress induces an abnormally high expression of miR-25 and miR-93 by activating the demethylase TET1 (Wu et al., 2017), which is a microRNA (miRNA) that directly targets the mRNA of genes within the STING pathway and blocks the transmission of immune signals at the transcriptional level. Meanwhile, the glycolytic end product lactate exerts systemic immunosuppression by reshaping the TME. Lactic acid not only directly reduces the catalytic efficiency of key enzymes, such as cGAS, by lowering pH (Wu et al., 2025a), but also promotes the overexpression of the nucleic acid-degrading enzyme TREX2 by reducing acidotic stress, thereby eliminating activation signals from the substrate level. This multi-method inhibition pathway, comprising “an acidic environment, enzyme activity inhibition, and nucleic acid degradation,” provides a stable metabolic barrier for cancer progression.

In addition to glucose metabolism, the remodeling of lipid and amino acid metabolism also induces dual immune effects via STING (Shen et al., 2024). This suggests that the activation state of cGAS-STING is essentially the result of the combined effects of changes in intracellular metabolic flux abundance and extracellular biochemical stress.

### 4.3 Immunosensitization of tumor microbial components

The tumor microbiome not only serves as a natural immune modulator but also directly regulates the cGAS-STING signaling pathway. Its mechanisms of action encompass multiple dimensions, including ion-assisted, nucleotide-induced, and biomimetic engineering interventions.

First, metal ions act as catalysts for microbial regulation of cGAS activity. Studies have shown that Mn^2+^ can directly bind to cGAS, enhancing its enzymatic activity and sensitivity to dsDNA, thereby strengthening antitumor immunity at the systemic level (Wu et al., 2025a; Huang and Zhou, 2023).

Second, bacteriogenic nucleotides can act as direct agonists of the STING pathway. For example, cyclic diadenosine monophosphate (c-di-AMP) can directly activate STING and induce the IFN signaling pathway, promoting M1 macrophage polarization and enhancing the antigen-presenting function in dendritic cells.

Furthermore, bioengineering techniques have enabled precise regulation of this activation process. Li et al. (2025) developed bio-mineralized engineered bacterial outer membrane vesicle (OMV)–ECL-expressing lactate dehydrogenase. In an acidic lysosomal environment, this carrier releases Ca^2+^ and Mn^2+^, and the OMVs, in synergy with Mn^2+^, induce the extensive phosphorylation of STING, leading to the release of Type I interferons and an enhanced immune response. Simultaneously, the loaded enzyme consumes lactate in the TME, thereby indirectly lifting the immunosuppressive barrier imposed by high glycolysis Li et al. (2025a). This study provides a specific mechanism at the molecular level for activation of the STING pathway via microbial components, opening up new directions for research on microbiotic–immune interactions.

### 4.4 Cascading regulation of the “microorganisms–metabolism–immunity” axis

Based on the current evidence, a multidimensional interplay has emerged, which can be conceptualized as the “microbiota–metabolism–immunity” axis. The tumor microbiome enhances glycolytic activity by producing metabolic byproducts such as lactate, which upregulate the expression of key glycolytic enzymes, such as PKM2 and HK2, in cancer cells. This process not only accelerates glucose consumption but also leads to lactate accumulation, creating an acidic microenvironment. As glycolytic flux increases, the tumor metabolism shifts toward immunosuppression, resulting in a “cold tumor” state. Activated ADSL enzymes produce fumarate in the endoplasmic reticulum, competitively occupying the STING activation site, thereby inhibiting STING pathway activity and causing the tumor to enter an immunologically inert state (Duan et al., 2025).

Meanwhile, high glucose promotes glucose binding to the NSUN2 protein, thereby stabilizing TREX2 mRNA and increasing the expression of TREX2, which is a cytoplasmic nucleolytic enzyme that clears the dsDNA substrates required for STING activation. This makes it difficult for immune cells such as CD8^+^ T cells to recognize tumor cells, and these molecular events directly affect the activity of the cGAS-STING pathway.

**FIGURE 1:**
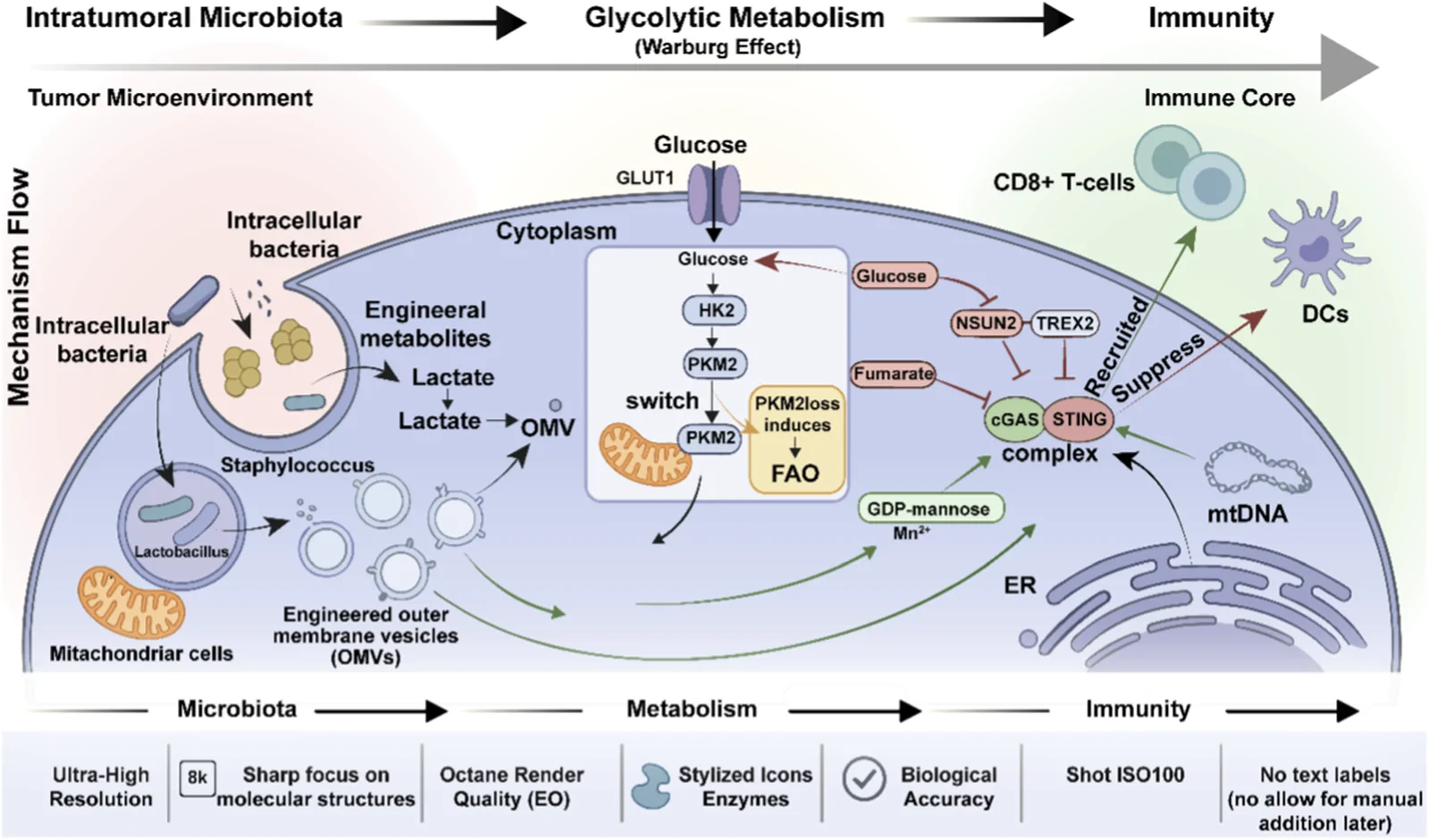
The “microbiome–metabolism–immunity” regulatory network in breast cancer. This mechanistic model illustrates the intricate intracellular crosstalk between intratumoral microbiota, glycolytic reprogramming, and the cGAS-STING signaling complex. Intratumoral bacteria and engineered OMVs modulate glycolytic enzymes (by influencing glycolytic activity) and alter metabolic flux. Glycolytic intermediates exert dual regulatory effects on immunity: glucose-driven activation of NSUN2/TREX2 and fumarate derived from ADSL competitively inhibit the cGAS-STING complex, thereby promoting immunosuppression. In contrast, metabolic stress-induced mtDNA leakage, GDP-mannose, and bacteria-derived manganese ions synergistically activate cGAS-STING, facilitating CD8^+^ T cell recruitment and remodeling the TME. Note: ADSL, adenylosuccinate lyase; cGAS, cyclic GMP-AMP synthase; DCs, dendritic cells; ER, endoplasmic reticulum; FAO, fatty acid oxidation; GDP-mannose, guanosine diphosphate-mannose; GLUT1, glucose transporter 1; HK2, hexokinase 2; mtDNA, mitochondrial DNA; NSUN2, NOP2/Sun RNA methyltransferase 2; OMVs, engineered bacterial outer membrane vesicles; PKM2, pyruvate kinase M2; STING, stimulator of interferon genes; TME, tumor microenvironment; TREX2, three-prime repair exonuclease 2.

However, immune suppression is not irreversible (Liu et al., 2024b). Li et al. (2025a) provided key evidence for reversing the immune status of cold tumors: Mn^2+^ released from engineered bacterial outer membrane vesicles can directly enhance cGAS sensitivity to DNA. Concurrently, lactate dehydrogenase loaded onto the carrier clears lactate from the microenvironment, thereby alleviating immune suppression caused by acid stress. Building on this, mitochondrial damage caused by impaired glycolysis leads to the release of mtDNA, which synergistically triggers the acute activation of STING, induces the release of Type I interferons, and promotes the recruitment and infiltration of killer T cells, transforming the TME from “cold” to “hot” (Ishikawa and Barber, 2008; Sun et al., 2013). Therefore, the immunological fate of breast cancer is not static but depends on the microbiome-guided metabolic flux of cancer cells and the regulation of the cGAS-STING pathway by metabolites (Wu et al., 2025b). The regulatory mechanism of the cGAS-STING pathway is depicted in Figure 1.

### 4.5 Multimodal combination therapy for clinical translation

It is worth noting that microorganisms influence the production and release of cytokines by generating metabolites and modulating immune and inflammatory responses, thereby regulating tumor initiation, metastasis, and treatment response. Conversely, cytokines can influence the microenvironment and tumor behavior by altering microbial activity within the TME. For example, IFN can help shape the immune microenvironment and eliminate tumor cells through various forms of programmed cell death (PCD), such as apoptosis, autophagy, pyroptosis, and ferroptosis (Yao et al., 2025). At the same time, factors such as changes in the local immune environment, conditions in the tumor microenvironment (such as hypoxia and low pH), and host-specific characteristics (such as genetics and lifestyle) all influence the abundance and structure of the tumor microbiome (Li et al., 2026). This bidirectional regulation suggests that the relationship between microbes, the immune system, and metabolism is not a simple linear one. This finding provides a new theoretical foundation for therapeutic strategies targeting the “microbiome–metabolism–immune” axis.

#### 4.5.1 Microbial recombination and signal induction

The aforementioned “microbial–metabolism–immunity” cascade model reveals the complex regulatory networks within the breast cancer microenvironment. The therapeutic paradigm for breast cancer is undergoing a fundamental shift, transitioning from isolated cytotoxic interventions toward a multidimensional, system-level remodeling of the TME. Interventions targeting the root causes of the “microbe–metabolism–immunity” axis focus on activating substrates and eliminating harmful colonizing bacteria in the cGAS-STING pathway.

Reregulating the intratumoral microbiome using antibiotics, probiotics, or engineered bacteria is a primary strategy for reshaping the immune microenvironment. The targeted elimination of intracellular colonization and the introduction of specific extracellular bacteria can effectively weaken the immunosuppressive barrier and prevent postoperative metastasis and recurrence (Fu et al., 2022; Yao et al., 2026). As novel STING nano-agonists, OMVs synergistically modulate lactate metabolism and enhance the efficacy of antitumor immunotherapy (Toyofuku et al., 2019).

Using the sustained accumulation of DNA damage and nanoscale sensitization as therapeutic strategies, activation of the cGAS-STING pathway by specific metabolites represents a novel approach to enhancing immunotherapy. Studies have confirmed that guanosine diphosphate mannose (GDP-mannose) specifically blocks the homologous recombination (HR) repair pathway using BRCA2 proteins, thereby dynamically interfering with protein and genomic stability. This metabolic intervention induces DNA damage and mitochondrial DNA (mtDNA) accumulation (Yang et al., 2022; Han et al., 2020), significantly activating downstream STING signals, and plays a dominant regulatory role in the immunotherapy of TNBC. Zhong et al. (2025) developed a manganese-based, porous silica nanocarrier that targets mitochondria. By precisely inducing the release of mtDNA in synergy with Mn^2+^ to activate the cGAS-STING pathway, this approach significantly enhanced the therapeutic efficacy of anti-PD-L1therapy in TNBC patients (Li and Chen, 2018).

#### 4.5.2 Metabolic interception and multimodal collaboration

After immune-sensing, it is necessary to overcome the tumor’s defensive barriers through multidimensional metabolic interventions and multimodal approaches to extend the therapeutic scope from tumor cells to effector T cells to restore their metabolic vitality. Enhancing the glycolysis level of these cells activates the cross-organelle metabolism–immune feedback mechanism. From the perspective of effector T cells, Shen et al. (2024) proposed that the increase in glycolytic flux induced by HIF-1α activation significantly promotes Interferon-γ (IFN-γ) synthesis. This metabolic stress-induced immune activation effect provides a direction for the development of novel metabolic–immune combined intervention strategies (Zhong et al., 2025).

In view of TNBC’s high dependence on glycolytic metabolism, targeting the glycolytic axis and inducing metabolic synthetic lethality are particularly critical. The metabolic fitness of tumor cells can be effectively inhibited by mimicking a fasting diet, targeting the glucose–NSUN2–TREX2 axis, and using inhibitors of key glycolytic enzymes such as HK2 and PKM2. It is worth noting that inhibiting glycolysis alone may induce metabolic compensation in cancer cells toward the FAO pathway. In such cases, the combined use of EZH2 and FAO inhibitors can induce a metabolic crisis in cancer cells, thereby eliminating TNBC cells that have undergone metabolic conversion (Zhang et al., 2024).

Microbial metabolites and metabolic inhibitors can exert synergistic effects. Studies have confirmed that butyrate can induce the expression of the monocarboxylic acid transporter MCT4 in breast cancer cells, thereby significantly enhancing the antitumor activity of the rate-limiting enzyme inhibitor 3-bromopyruvate (3-BP). This finding supports the “microbiome–metabolism” combined intervention model, which utilizes microbial metabolites to sensitize tumor cells and thereby enhance the clinical efficacy of glycolysis-targeted drugs (Queirós et al., 2012).

As a central hub connecting innate and adaptive immune responses, the cGAS-STING pathway plays a critical role in antitumor immunity and has emerged as a promising target for cancer treatment (Chen et al., 2021; Luo et al., 2022). The immunosuppressive characteristics of TNBC, the development of novel STING small-molecule agonists and immunosuppressants, and the use of nanocarriers to achieve precise delivery in space and time are areas that require urgent attention (Lai et al., 2021). This will induce mtDNA release from mitochondria and recruit killer immune cells such as CD8^+^T cells, combined with immune checkpoint blockades (such as anti-PD-L1 therapy) or peptide blockades of ADSL translocation (Li and Chen, 2018; Cheng et al., 2023). In addition, synergistic enhancement of cGAS sensitivity with metal Mn^2+^ ions holds promise for targeted activation and desensitization, comprehensively remodeling the TME, and overcoming the bottlenecks currently faced by monotherapy. To clearly define these emerging treatment models, Table 1 provides a comprehensive summary of targeted therapies, microbial interventions, and multi-modal combination treatment strategies for the breast cancer metabolism-immune axis.

## 5 Summary and prospects

This study systematically elucidates the complex interactions and regulatory mechanisms of glycolysis, the cGAS-STING pathway, and the tumor microbiome in the progression of breast cancer. Current evidence suggests that these three components exist in a dynamic equilibrium within the TME: glycolysis not only provides the tumor with energy and substrates for biosynthesis but also remodels cancer phenotypes and induces treatment resistance through metabolic reprogramming; the cGAS-STING pathway has a dual function, enhancing antitumor immunity while also promoting the formation of an immunosuppressive microenvironment; and the tumor microbiota and their metabolites affect tumor metastatic potential and immune status through direct bacteria–cancer cell interactions.

Crucially, these three core elements do not exist in isolation but are dynamically intertwined through a bidirectional closed-loop mechanism. On the one hand, they are tightly coupled through a cascade of “microbiome sensing—metabolic flux—signal transduction.” The tumor microbiome can reshape glycolytic metabolism, thereby regulating the cGAS-STING pathway. On the other hand, the massive release of IFN-I and pro-inflammatory cytokines triggered by potent STING activation profoundly affects the tumor microenvironment and alters the richness of the intratumoral microbiome. This bidirectional regulation ultimately determines the immune evolutionary trajectory and treatment response of breast cancer; this finding provides a new perspective on understanding the onset and progression of breast cancer.

## Glossary

ADSL: adenylosuccinate lyase
AMPK: AMP-activated protein kinase
BRCA2: breast cancer susceptibility protein 2
CDNs: cyclic dinucleotides
cGAS: cyclic GMP-AMP synthase
DCs: dendritic cells
EMT: epithelial–mesenchymal transition
EZH2: enhancer of zeste homolog 2
FAO: fatty acid oxidation
FMD: fasting-mimicking diet
G6PI: glucose-6-phosphate isomerase
GDP-mannose: guanosine diphosphate-mannose
GLS: glutaminase
GLUT1: glucose transporter 1
HDAC: histone deacetylase
HER2: human epidermal growth factor receptor 2
HIF-1α: hypoxia-inducible factor 1α
HK2: hexokinase 2
HR: homologous recombination
IFN: interferon
LDHA: lactate dehydrogenase A
lncRNA: long non-coding RNA
MCT4: monocarboxylate transporter 4
MDSCs: myeloid-derived suppressor cells
mtDNA: mitochondrial DNA
NSUN2: NOP2/Sun RNA methyltransferase 2
OMVs: engineered bacterial outer membrane vesicles
PFKFB3: 6-phosphofructo-2-kinase/fructose-2,6-biphosphatase 3
PKM2: pyruvate kinase M2
PSAT1: phosphoserine aminotransferase 1
ROS: reactive oxygen species
STING: stimulator of interferon genes
TAMs: tumor-associated macrophages
TILs: tumor-infiltrating lymphocytes
TMB: tumor mutation burden
TME: tumor microenvironment
TNBC: triple-negative breast cancer
Tregs: regulatory T cells
TREX2: three-prime repair exonuclease 2
